# Changes in grassland soil types lead to different characteristics of bacterial and fungal communities in Northwest Liaoning, China

**DOI:** 10.3389/fmicb.2023.1205574

**Published:** 2023-06-28

**Authors:** Xinwei Ma, Baihui Ren, Jianxin Yu, Jiayu Wang, Long Bai, Jiahuan Li, Daiyan Li, Meng Meng

**Affiliations:** College of Horticulture, Shenyang Agricultural University, Shenyang, Liaoning, China

**Keywords:** grassland soil type, fungal community, bacterial community, microbial diversity, microbial community composition, environmental factors

## Abstract

**Introduction:**

Soil microbial communities are critical in regulating grassland biogeochemical cycles and ecosystem functions, but the mechanisms of how environmental factors affect changes in the structural composition and diversity of soil microbial communities in different grassland soil types is not fully understood in northwest Liaoning, China.

**Methods:**

We investigated the characteristics and drivers of bacterial and fungal communities in 4 grassland soil types with 11 sites across this region using high-throughput Illumina sequencing.

**Results and Discussion:**

Actinobacteria and Ascomycota were the dominant phyla of bacterial and fungal communities, respectively, but their relative abundances were not significantly different among different grassland soil types. The abundance, number of OTUs, number of species and diversity of both bacterial and fungal communities in warm and temperate ecotone soil were the highest, while the warm-temperate shrub soil had the lowest microbial diversity. Besides, environmental factors were not significantly correlated with soil bacterial Alpha diversity index. However, there was a highly significant negative correlation between soil pH and Shannon index of fungal communities, and a highly significant positive correlation between plant cover and Chao1 index as well as Observed species of fungal communities. Analysis of similarities showed that the structural composition of microbial communities differed significantly among different grassland soil types. Meanwhile, the microbial community structure of temperate steppe-sandy soil was significantly different from that of other grassland soil types. Redundancy analysis revealed that soil total nitrogen content, pH and conductivity were important influencing factors causing changes in soil bacterial communities, while soil organic carbon, total nitrogen content and conductivity mainly drove the differentiation of soil fungal communities. In addition, the degree of connection in the soil bacterial network of grassland was much higher than that in the fungal network and soil bacterial and fungal communities were inconsistently limited by environmental factors. Our results showed that the microbial community structure, composition and diversity of different grassland soil types in northwest Liaoning differed significantly and were significantly influenced by environmental factors. Microbial community structure and the observation of soil total nitrogen and organic carbon content can predict the health changes of grassland ecosystems to a certain extent.

## 1. Introduction

Grasslands are one of the most widespread vegetation types in the world, covering about 1/3 of the land area (Kemp et al., 2013), and are an important part of terrestrial ecosystems (Merbold et al., 2014). Grassland types are divided into nine major types (Wu, 1990), temperature and precipitation are often used in the study of grassland type classification (Del Vecchio et al., 2018; Peng et al., 2021). The climate of northwestern Liaoning Province is transitional from temperate semi-humid to semi-arid from southeast to northwest, and is characterized by abundant sunshine, rain and heat in the same season, high cumulative temperature, and low precipitation (Wang et al., 2013). The land in this area is barren, with serious soil erosion and desertification (Wang et al., 2021). Under the combined influence, many complex grassland soil types have been formed, among which the temperate steppe and the warm-temperate shrub are the most unique and have great research value.

With the in-depth research and practice, many researchers have realized that the degradation of grassland is not only the change of surface vegetation and soil physicochemical properties, but also the change of grassland soil microbial community structure and diversity (Yu et al., 2021; Wang et al., 2022). Soil is rich in living microbiota, which are an important part of soil ecosystem. The species, quantity, distribution, life activity pattern of microbial communities and the transformation with the materials and energy in the soil are the values of soil microbial resources. It plays a leading role in ecosystem functions such as soil organic matter decomposition and nutrient cycling (Wei et al., 2017). Compared with soil microorganisms such as fungi, actinomycetes and soil protozoa, soil bacteria are the most important taxon, accounting for about 70 to 90% of the total soil microorganisms, and play a dominant role in biogeochemical cycling processes (Van der Heijden et al., 2008; Bar-On et al., 2018). However, fungi have higher relative abundance and diversity in the rhizosphere soil (Bahram et al., 2021). Soil bacteria and fungi together drive and regulate biological processes such as mineralization, degradation and transformation of various nutrient element in the soil, and also have an important impact on plant growth and biological yield (Kaiser et al., 2016). They are directly participate in biochemical processes such as ammonification, nitrification, denitrification and biofixation of nitrogen in the soil (Huang et al., 2013), and play a key role in soil organic matter degradation and soil carbon cycling processes (Chen and Sinsabaugh, 2020). Thus, the composition and biodiversity of soil bacterial and fungal communities in grassland ecosystems have become the focus of attention by many scholars (Bhattacharyya et al., 2014).

The construction process of microbial communities is decisively influenced by environmental factors (Tatari et al., 2017). Soil microorganisms are very sensitive to the living microenvironment and can respond rapidly to changes in the soil environment, thereby affecting the species diversity of plant community and the formation of soil structure. Therefore, changes in soil microbial community structure and diversity can be used as important indicator to measure the health changes of grassland ecosystems and the degree of grassland degradation or restoration (Wu et al., 2014). Tardy et al. (2014) showed that the biodiversity of soil bacterial community determined the structure of soil bacterial community and its ecological service functions, while the study by Jia et al. (2020) showed that environmental factors play a very important role in driving fungal community construction. Existing studies have shown that soil microbial diversity differed significantly among different grassland community type (Qi et al., 2021), and the more suitable the habitat conditions (High total nitrogen and organic carbon content and suitable soil pH, etc.), the higher the diversity was Santamaría et al. (Santamaría et al., 2018). Besides, the diversity of different soil bacterial communities was mainly influenced by pH, organic matter, alkaline nitrogen and effective phosphorus content (Dunbar et al., 2002; Roy et al., 2020; Jiang et al., 2021), while soil pH and organic matter content played a decisive role in the structure of different soil fungal communities (Guo et al., 2021; Shang et al., 2021). Therefore, the microbial community structure and diversity could differ under different grassland soil types and may be influenced by distinct environmental factors. Further studies to analyze the bacterial and fungal communities and their diversity in different regional grassland soil types are conducive to better utilization and development of grassland resources.

Although many studies have been conducted on the influence of environment on microbial communities, against the background of relatively limited overall knowledge of soil microorganisms in different grassland types in northwest Liaoning, the study on the characteristics of soil microbial communities and their influencing factors in different grassland types in northwest Liaoning can not only enrich theoretically the research content of different grassland types, but also provide scientific basis for maintaining the stability of fragile ecosystems in our province and protecting the diversity of regional biological resources. To understand the characteristics and influencing factors of bacterial and fungal communities in different grassland soil types in northwest Liaoning, we used high-throughput sequencing to answer two questions: (1) Are there differences in the structural composition of bacterial and fungal communities in different grassland soil types? and (2) What are the underlying mechanisms for the effects of environmental factors on soil bacterial and fungal communities? Based on the results of previous studies, we hypothesize that: (i) There could be significant differences in the structural composition of the bacterial and fungal communities in different grassland soil types and (ii) Changes in environmental factors such as soil total nitrogen (TN) and soil organic carbon (SOC) would drive the observed changes in soil bacterial and fungal communities.

## 2. Materials and methods

### 2.1. Site description

The climate type of northwest Liaoning is semi-arid monsoon continental climate with dry and rainless. Moreover, the more it tends to the center of the continent, the more arid it becomes, and the annual and daily differences in temperature also become bigger. Vegetation also transitions from forest to grassland and desert. The average annual precipitation is about 562 mm, the average annual temperature is 7.4°C, the average frost-free period is 160 d, the accumulated temperature ≥ 10°C is 3,321 ~ 3,532°C, the annual sunshine hours are 2,823 ~ 2,944 h, the annual solar radiation is 5,719 ~ 6,050 MJ/m^2^. Due to the influence of monsoon, precipitation is generally concentrated in summer, accounting for about 75% of the annual precipitation, and the maximum possible evaporation is 1,121 mm, which makes water resources scarce. The wind is mostly northwesterly and southeasterly, with a high wind volume, greater than 5 m/s, and nearly 50 d of sandy days in a year (Tong, 2012). Grassland plants in northwest Liaoning are mainly dry herbaceous plants, and the grass species with high frequency are: *Cleistogenes squarrosa* (Trin.) Keng, *Stipa grandis* P.A. Smirn., *Lespedeza bicolor* Turcz., *Agropyron cristatum* (L.) Gaertn., *Cleistogenes polyphylla* Keng ex Keng f. et L. Liou, *Cleistogenes caespitosa* Keng, *Potentilla chinensis* Ser., and shrubs are mainly *Vitex negundo* L. var. *het*erophylla (Franch.) Rehd., with very high depression, even >60% in some places.

### 2.2. Sample collection and soil physicochemical analysis

Sampling was conducted during the growing season (August 2019) at 11 sites selected from four grassland soil types: temperate steppe-sandy soil (TS1), temperate steppe-loamy soil (TS2), warm and temperate ecotone (ecotone) and warm-temperate shrub (WST). Three 1 m × 1 m plots were randomly set up as replicates at each site, and soil was collected from the 0–20 cm layer using a five-point sampling method, and plant samples were collected to obtain information on plant composition, cover, and biomass. This study did not involve endangered or protected species and complied with relevant institutional, national and international guidelines and laws, and no genotyping data were analyzed or generated during the study.

All samples were cooled with ice packs and transported to the laboratory immediately. After thorough homogenization, the composite soils were sieved (100 mesh) to remove fine roots and large organic debris and divided into two portions. One portion was stored at 4°C for characterization of soil physical and chemical properties, and the other portion was stored at −80°C for DNA extraction. Soil physicochemical property analyses (total soil nitrogen, total phosphorus, organic carbon, pH, conductivity, alkaline soluble nitrogen, ammonium nitrogen, nitrate nitrogen, fast-acting phosphorus, organic nitrogen, and plant biomass) were performed according to Bao (2000) and the basic sample information is shown in Table 1.

**Table 1:** Summary of basic information of sample plots.

Site	Grassland soil type	Latitude and longitude	Soil texture	Dominant plant	TPC (%)	TPB (g∙m^−2^)
P1	TS1	N 42°48ˊ, E122°32ˊ	Sand	*Cleistogenes squarrosa* (Trin.) Keng	47%	116.13
P2	TS1	N 42°55ˊ, E122°18ˊ	Sand	*Cleistogenes squarrosa* (Trin.) Keng	53%	169.87
P3	TS1	N 42°49ˊ, E122°2ˊ	Sand	*Cleistogenes squarrosa* (Trin.) Keng	43%	240.77
P4	TS2	N 42°25ˊ, E121°30ˊ	Loam	*Cleistogenes polyphylla* Keng ex Keng f. et L. Liou	68%	127.87
P5	Ecotone	N 42°3ˊ, E121°2ˊ	Loam	*Cleistogenes caespitosa* Keng	67%	124.97
P6	WST	N 42°3ˊ, E120°5ˊ	Loam	*Cleistogenes polyphylla* Keng ex Keng f. et L. Liou	50%	87.83
P7	TS2	N 42°14ˊ, E120°44ˊ	Loam	*Cleistogenes squarrosa* (Trin.) Keng	53%	85.27
P8	Ecotone	N 42°10ˊ, E120°55ˊ	Loam	*Cleistogenes polyphylla* Keng ex Keng f. et L. Liou	48%	97.50
P9	TS2	N 41°56ˊ, E119°47ˊ	Loam	*Leymus chinensis* (Trin.) Tzvel.	28%	110.90
P10	WST	N 41°18ˊ, E119°49ˊ	Loam	*Themeda japonica* (Willd.) Tanaka.	45%	153.46
P11	WST	N 42°12ˊ, E122°2ˊ	Loam	*Cleistogenes caespitosa* Keng	53%	63.93

TS1 represents temperate steppe-sandy soil; TS2 represents temperate steppe-loamy soil; Ecotone represents warm and temperate ecotone; WST represents warm-temperate shrub; TPC represents total plant cover; TPB represents total plant biomass.

### 2.3. Illumina sequencing

The Power Soil DNA Isolation Kit (MoBio, United States) was selected to extract genomic DNA from the samples, after which the purity and concentration of DNA were tested using agarose gel electrophoresis and Nanodrop. The diluted genomic DNA was used as template for PCR using specific primers with Barcode and efficient high fidelity enzymes according to the selection of sequencing regions. We used the 515F (5’-GTGCCAGCMGCCGCGGTAA-3′)/909R (5’-CCCCGYCAATTCMTTTRAGT-3′) for bacterial amplification primers, and the fungal amplification primer was ITS4 (5’-TCCTCCGCTTATTGATATGC-3′)/gITS7F (5’-GTGARTCATCGA RTCTTTG-3′) (Geng et al., 2022). Library construction was performed using TruSeq® DNA PCR-Free Sample Preparation Kit library construction kit. The constructed libraries were quantified by Qubit and Qpcr. After the libraries were qualified, the v2 sequencing kit (2 × 250 bp) and Miseq sequencer were used for on-board sequencing. The resultant PCR products were combined at equimolar concentrations before being sequenced on an lllumina Miseq platform at GOOAL GENE.

### 2.4. Processing of sequencing data

Acquired initial sequence used the Quantitative Insights into Microbial Ecology (QIIME v.1.9.1) quality control process to conduct the extraction of high-quality clean tags (Bolyen et al., 2018),^1^ the sequences were clustered into operational taxonomic units (OTUs) according to 97% pairwise identity with the USEARCH tool based on the UCHIME algorithm (Edgar et al., 2011). The following databases were used as a reference for taxonomic assignments: 16S rRNA Greengenes (McDonald et al., 2012) (version 13.8) for bacteria and UNITE (Abarenkov et al., 2010) (version 12_11) for fungi. Alpha-diversity metrics [Chao1 (Chao, 1984), Observed species, Shannon (Shannon, 1948), Simpson (Simpson, 1949), Pielou’s evenness (Pielou, 1966) and Good’s coverage (Good, 1953)], beta diversity metrics (Bray–Curtis dissimilarity) were estimated using the diversity plugin with samples were rarefied to sequences. The Rarefaction method was used for Alpha and Beta diversity analysis and abundance difference analysis. In the analysis of abundance difference and Beta diversity, the pumping depth was set at 95% of the lowest sample sequence size. For Alpha diversity analysis, the minimum drawing depth was set at 10, and then 10 depth values were evenly selected between 95% of the minimum sequencing depth in all samples. Each depth value was drawn 10 times to calculate the above Alpha diversity index. By default, the average score at the maximum drawing depth was selected as the alpha diversity index.

The DNA sequences in this study have been deposited in the National Center for Biotechnology Information (NCBI) Sequence Read Archive (SRA) database under accession number PRJNA922357.

### 2.5. Statistical analyses

One-way analysis of variance (ANOVA) was performed using the software SPSS22.0 (IBM Co, Armonk, NY, United States) to determine differences in soil physicochemical properties among different grassland soil types. Kruskal-wallis test was used to determine differences in the relative abundance of microbial communities at the taxonomic level of phylum and genus. Pearson correlation analysis was performed to explore the correlation between environmental factors and soil microbial diversity. The significance between different treatments was confirmed using Tukey’s HSD test at *p* < 0.05.

The “ggplot2” package in R was used to draw box line plots and to visualize the compositional distribution of each sample at the systematic taxonomic level to determine differences in microbial community composition and alpha diversity among different grassland soil types. The structural composition of the microbial communities of the different grassland soil types was visualized using principal coordinate analysis (PCoA) using the “ape” package in R language, which is based on the Bray-Curtis heterogeneity matrix. To examine the effect of grassland soil type on microbial β-diversity, Anosim (Analysis of similarities) analysis was used. Also, the SparCC algorithm was used together with the “ggraph” package and igraph to construct and map symbiotic networks to observe differences in bacterial and fungal communities (Filter out ASV/OTU with less than 10 total sequences and less than 5 samples). The effects of environmental factors on microbial community structure were analyzed by constrained ordinal redundancy analysis (RDA) in CANOCO 5.0 software (Microcomputer Power, Ithaca, NY, United States). The effect of each variable was assessed using an RDA-based Monte Carlo test (999 permutations).

## 3. Results

### 3.1. Soil physicochemical properties of different grassland soil types

The soil physicochemical properties of different grassland soil types are shown in Table 2. Soil organic carbon, ammonium nitrogen, nitrate nitrogen and electrical conductivity were significantly differences among different grassland soil types (*p* < 0.05). Warm and temperate ecotone soils had the highest total nitrogen (1.59 g/kg), total phosphorus (0.43 g/kg), organic carbon (11.33 g/kg) and ammonium nitrogen contents (11.82 mg/kg), while the lowest pH value (6.33). The warm-temperate shrub soils had the highest soil conductivity (46.46 S/m) and nitrate nitrogen content (10.56 mg/kg), and the temperature steppe-sandy soils had the lowest soil contents of total nitrogen (0.95 g/kg), total phosphorus (0.27 g/kg), organic carbon (3.43 g/kg), electrical conductivity (21.67 S/m), ammonium nitrogen (6.63 mg/kg) and nitrate nitrogen (3.36 mg/kg). The pH range of all soil samples was in the range of 6–7, which were slightly acidic.

**Table 2:** Soil physicochemical properties in different grassland soil types.

	TN (g∙kg^−1^)	TP (g∙kg^−1^)	SOC (g∙kg^−1^)	pH	EC (S∙m^−1^)	NH_4_^+^-N (mg∙kg^−1^)	NO_3_^−^N (mg∙kg^−1^)
TS1	0.95 ± 0.08	0.27 ± 0.08	3.43 ± 0.34b	6.70 ± 0.08	21.67 ± 1.31b	6.63 ± 0.97b	3.36 ± 0.38c
TS2	1.46 ± 0.13	0.28 ± 0.05	9.14 ± 0.86a	6.80 ± 0.25	47.90 ± 8.07a	8.44 ± 0.70ab	6.96 ± 2.25b
Ecotone	1.59 ± 0.42	0.43 ± 0.08	11.33 ± 0.69a	6.33 ± 0.09	38.46 ± 4.48ab	11.82 ± 1.21a	8.37 ± 1.79b
WST	1.30 ± 0.21	0.40 ± 0.07	11.21 ± 0.62a	6.94 ± 0.14	46.46 ± 6.75ab	11.21 ± 1.05a	10.56 ± 2.46a

TN represents total nitrogen; TP represents total phosphorus; SOC represents soil organic carbon; EC represents electrical conductivity; NH_4_^+^-N represents ammonium nitrogen; NO_3_^−^N represents nitrate nitrogen. Different letters in the same column indicate significant difference at the 0.05 level.

### 3.2. High-throughput sequencing analysis of microbial communities in different grassland soil types

#### 3.2.1. Analysis of alpha diversity of bacterial and fungal communities in different grassland soil types

**Figure 1:**
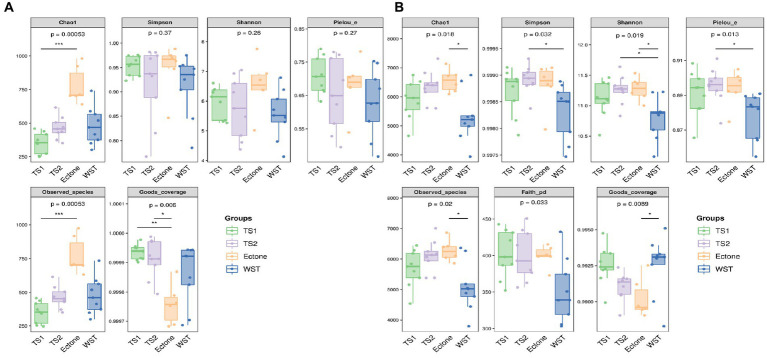
Box plot of Alpha diversity index for **(A)** bacterial and **(B)** fungal communities in different grassland soil types. TS1 represents temperate steppe-sandy soil; TS2 represents temperate steppe-loamy soil; Ecotone represents warm and temperate ecotone; WST represents warm-temperate shrub. The sample size of TS1, TS2 and WST is 9, and the sample size of Ecotone is 6.

As can be seen from the grouped boxplots in Figure 1, there existed significantly different of all the alpha diversity indicators of bacterial communities in different grassland soil types (*p* < 0.05), indicating that the abundance, number of species, diversity and evenness of soil bacterial communities in the four types were significantly different. Among the indicators of alpha diversity of fungal communities in different grassland soil types, the Chao1 index and the Observed species index were highly significantly different (*p* < 0.01), indicating that there were significant differences in the abundance and number of species of fungal communities in different grassland soil types, while the diversity and evenness were not significantly different (*p* > 0.05).

Among different grassland soil types, the order of Chao1 index and Observed species of bacterial communities were warm and temperate ecotone > temperate steppe-loamy soil > temperate steppe-sandy soil > warm-temperate shrub. It showed that the warm and temperate ecotone had the highest abundance of soil bacterial community, the largest number of soil bacterial OTUs and species. Meanwhile, the Shannon index was also highest in the temperate ecotone, indicating its high soil bacterial diversity. And the order of Simpson’s index and Pielou’s index were temperate steppe-loamy soil > warm and temperate ecotone > temperate steppe-sandy soil > warm-temperate shrub. It showed that the highest diversity and evenness of soil bacterial communities was in the temperate steppe-loamy soil. Good’s coverage (coverage of each sample library) was greater than 0.99, indicating that the sequencing results could fully reflect the real situation of microorganisms. The sequencing depth was sufficient to cover all the samples reflected by the rarefaction curve of Shannon diversity (Supplementary Figure S1A).

The order of Chao1 index and Observed species of fungal communities in different grassland soil types were warm and temperate ecotone > warm-temperate shrub > temperate steppe-loamy soil > temperate steppe-sandy soil. It showed that the highest abundance of soil fungal communities, the highest number of soil fungal OTUs and species, and high soil fungal diversity in the warm and temperate ecotone. Good’s coverage (coverage rate of each library) was greater than 0.9997, indicating that the sequencing results could fully reflect the real situation of microorganisms. The sequencing depth was sufficient to cover all the samples reflected by the rarefaction curve of Shannon diversity (Supplementary Figure S1B).

#### 3.2.2. Beta diversity analysis of bacterial and fungal communities in different grassland soil types

**Figure 2:**
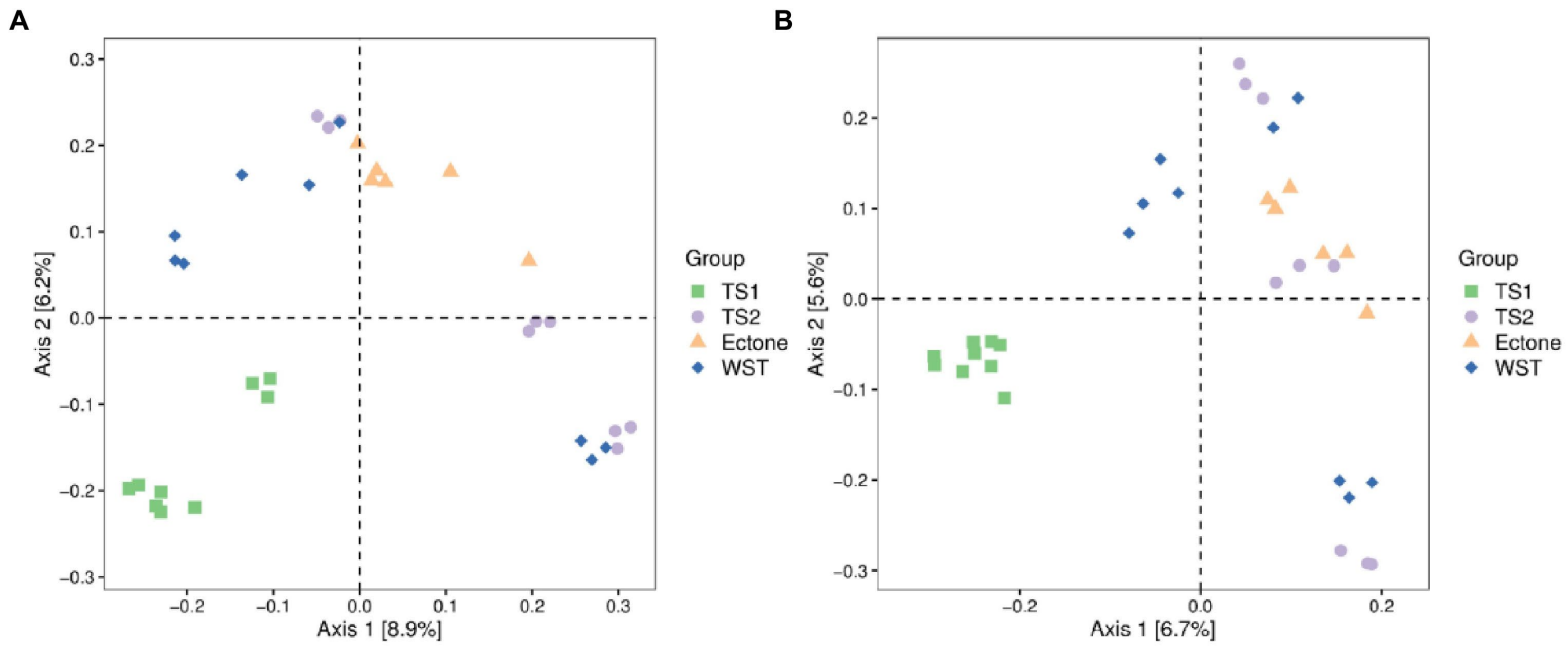
Principal coordinates analysis of **(A)** bacterial and **(B)** fungal communities in different grassland soil type. TS1 represents temperate steppe-sandy soil; TS2 represents temperate steppe-loamy soil; Ecotone represents warm and temperate ecotone; WST represents warm-temperate shrub. The sample size of TS1, TS2 and WST is 9, and the sample size of Ecotone is 6.

The sum of Axis1 and Axis2 explained 15.1 and 12.3% of the variation in soil bacterial and fungal communities, respectively (Figure 2). All samples were mainly concentrated in four different areas corresponding to the four different grassland soil types, demonstrating that the bacterial and fungal community structures of different grassland soil types were heterogeneous and had obvious regional characteristics. Among them, the distribution of bacterial communities among different grassland soil types was more independent and had obvious differences. While in the fungal community, only the samples of the temperate steppe-sandy soil were more concentrated and had good independence. The dispersion of the samples of the other three grassland soil types was high, and the structural composition of their respective fungal communities had some differences, but the species abundance and similarity were not obvious enough.

The cluster analysis revealed (Supplementary Figure S2) that the bacterial communities of the three grassland soil types were closely connected among the samples, except for the soil bacterial community of the warm-temperate shrub. Among them, the samples of temperate steppe-sandy soil had very short branches and high similarity among them. In contrast, among the soil fungal communities, only the samples of temperate steppe-sandy soil were closely connected and had the shortest branch length between samples and the highest similarity. This is consistent with the results of the principal coordinate analysis.

Anosim analysis showed that the structural composition of bacterial communities differed significantly among different grassland soil types (*p* < 0.05). While only the structural composition of fungal communities differed significantly between the temperate steppe-sandy soil and temperate steppe-loamy soil as well as the warm-temperate shrub, and between the warm and temperate ecotone and the warm-temperate shrub (*p* < 0.05). It proved that there was variability in the structural composition of bacterial and fungal communities among the four grassland soil types and verified with the results of PCoA analysis.

#### 3.2.3. Structural composition of bacterial and fungal communities in different grassland soil types

High-throughput sequencing yielded a total of 3,135,271 high quality sequences with an average length of 417.90 bp for bacteria, which were binned into 96,468 OTUs at 97% sequence identity, and a total of 3,905,574 high quality sequences with an average length of 231.82 bp for fungi, which were binned into 8,468 OTUs at 97% sequence identity.

**Figure 3:**
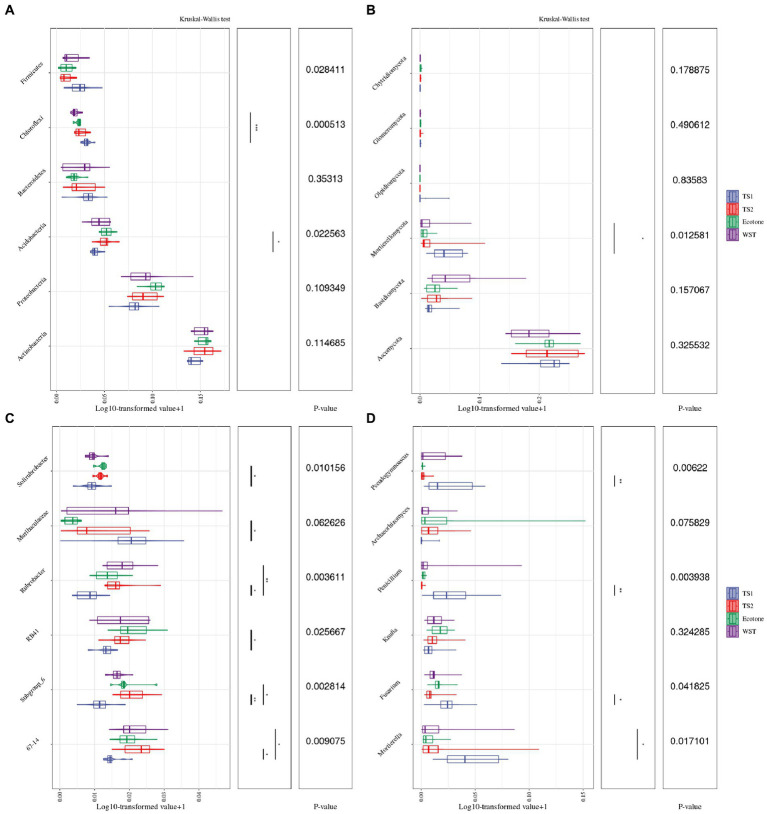
Differences in the top 6 relative abundance of microbial communities in different grassland soil types. **(A)** The relative abundance at the taxonomic level of the bacterial community phylum. **(B)** The relative abundance at the taxonomic level of the fungal community phylum. **(C)** The relative abundance at the taxonomic level of the bacterial community genus. **(D)** The relative abundance at the taxonomic level of the fungal community genus. The meanings of the upper and lower end lines, median and upper and lower edges of the boxes are the upper and lower quartile range (Interquartile range, IQR), median, maximum and minimum values (1.5 times the extreme value of the IQR range), respectively. *p*-values have been corrected for FDR multiple testing, and * represents the significance of the post-hoc tests between the two groups corresponding to the ends of the line, “*” means significant correlation (*p* < 0.05), “* *” means very significant correlation (*p* < 0.01).

The differences in the distribution of bacterial and fungal communities in different grassland soil types at the “phylum” taxonomic level are shown in Figures 3A,B. The dominant phyla of bacterial communities in all samples were *Actinobacteria*, *Proteobacteria* and *Acidobacteria*, with the average relative abundance of 41.39, 23.82 and 11.55%, respectively. Although *Actinobacteria* was the most dominant phylum of soil bacterial community shared by different grassland soil types, the differences in abundance among them were not significant. However, the relative abundance of *Acidobacteria* differed significantly among these grassland soil types (*p* < 0.05) and were both highest in warm and temperate ecotone (12.88%). The comparatively higher relative abundance of fungal communities were *Ascomycota*, *Basidiomycota* and *Mortierellomycota*, with average relative abundance of 62.55, 8.64 and 5.53%, respectively. *Ascomycota* was the most dominant phylum of soil fungal community shared by different grassland soil types, but the differences in abundance between them were not significant. Nevertheless, there were significant differences in the relative abundance of *Mortierellomycota* among these grassland soil types (*p* < 0.05). The highest relative number of *Mortierellomycota* was 11.15% in the temperate steppe-sandy soil.

Figures 1D, 3C show the differences in the distribution of bacterial and fungal communities in different grassland soil types at the taxonomic level of “genus.” *67–14*, *Subgroup 6*, *RB41*, *Rubrobacter*, *Muribaculaceae* and *Solirubrobacter* are the 6 genera with the highest relative abundance of bacterial communities, with the average relative abundance of 4.68, 4.00, 4.00, 3.44, 3.11 and 2.45%, respectively. *Mortierella*, *Fusarium*, *Knufia*, *Penicillium*, *Archaeorhizomyces* and *Pseudogymnoascus* were the 6 genera with the highest relative abundance in the fungal community, with the average relative abundance of 5.70, 3.84, 3.04, 3.01, 2.88 and 2.78%, respectively. They all differed significantly among different grassland soil types.

#### 3.2.4. Association network of soil bacterial and fungal communities in Northwest Liaoning grassland

**Figure 4:**
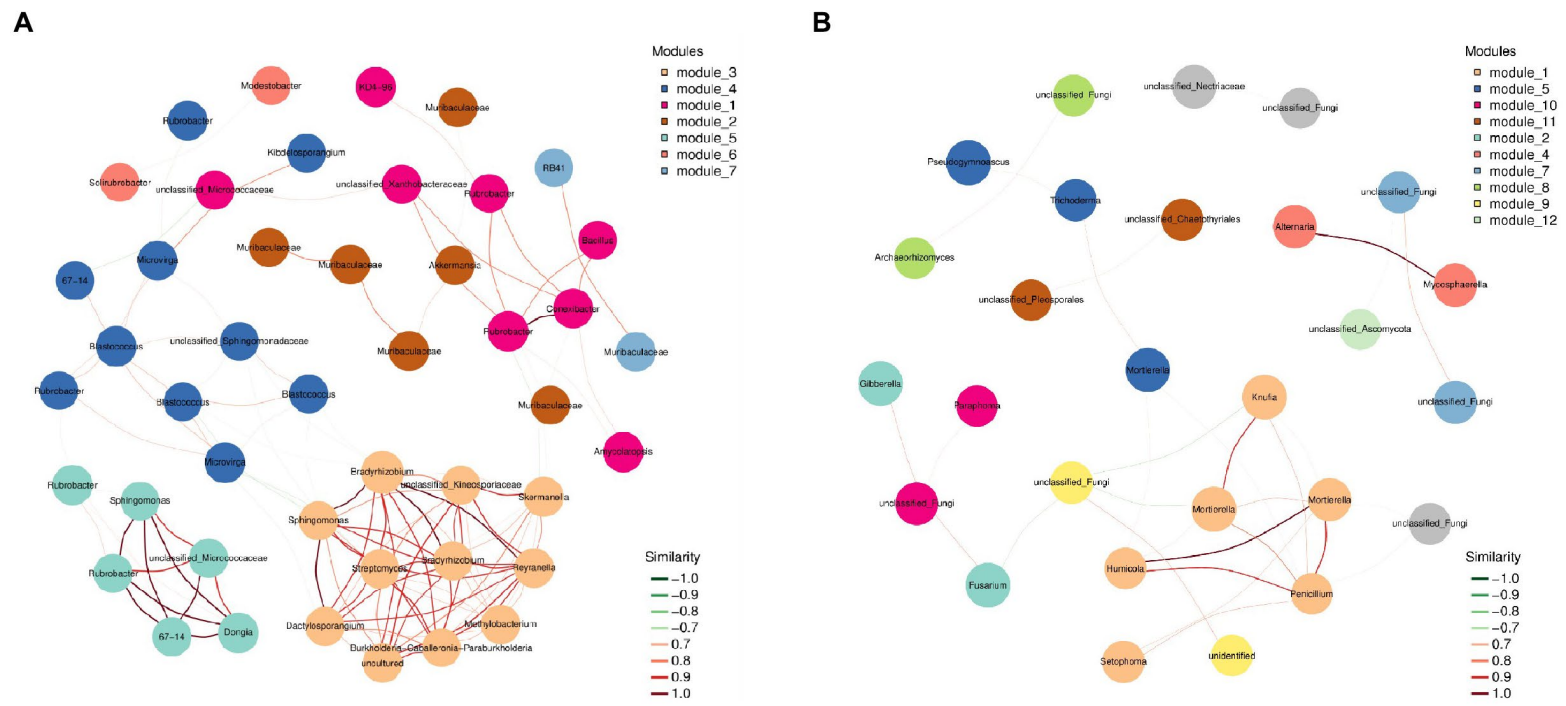
Network of **(A)** bacterial and **(B)** fungal communities in grasslands in Northwest Liaoning Province.

The species in the network diagram of soil bacterial communities in northwest Liaoning grassland were more active and formed more associations than the fungal communities network (Figure 4). In Table 3, the topological parameters of the network showed that the network of soil bacteria contained 1,568 nodes and 43,389 edges, while the network of soil fungi contained only 65 nodes and 97 edges. And the nodes of the network of soil bacteria were more closely connected than the network of soil fungi. Therefore, the northwest Liaoning grassland was mainly dominated by soil bacteria.

**Table 3:** Network topology parameters of soil bacterial and fungal communities.

	Average nearest neighbor degree	Average path lenghth	Number of vertice	Number of edge	Modularity
Bacterial communities	89.330	3.383	1,568	43,389	0.419
Fungal communities	4.229	3.495	65	97	0.557

### 3.3. Relationship between environmental factors and soil microbial communities

#### 3.3.1. Pearson correlation analysis between environmental factors and soil bacterial and fungal community diversity indices

**Figure 5:**
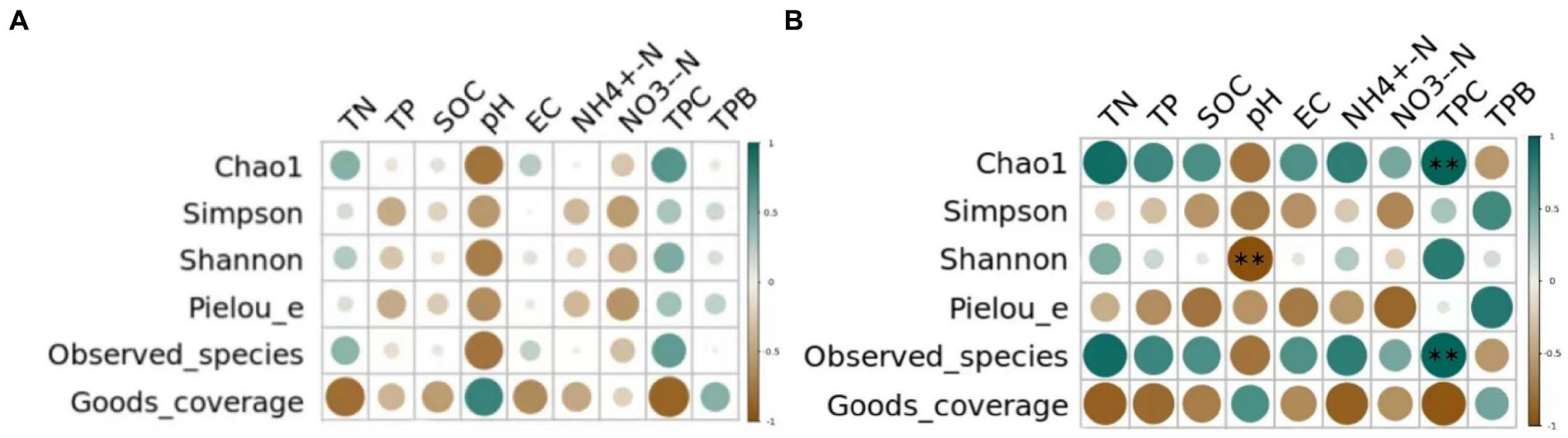
Correlation between environmental factors and soil **(A)** bacterial and **(B)** fungal communities’ diversity indexes. TN represents total nitrogen; TP represents total phosphorus; SOC represents soil organic carbon; EC represents electrical conductivity; NH4^+^-N represents ammonium nitrogen; NO3^−^-N represents nitrate nitrogen; TPC represents total plant cover; TPB represents total plant biomass. “*” means significant correlation (*p* < 0.05), “* *” means very significant correlation (*p* < 0.01).

Pearson correlation analysis was conducted between the diversity indices of bacterial and fungal communities of different grassland soil types and the underlying physicochemical properties. As shown in Figure 5 in the bacterial communities, the soil total nitrogen content, total phosphorus content, organic carbon content, pH, conductivity, ammonium nitrogen content, nitrate nitrogen content, plant cover and vegetation biomass were not significantly correlated with the Alpha diversity (*p* > 0.05). While in the fungal communities, a highly significant negative correlation existed between soil pH and Shannon index (*p* < 0.01) and plant cover and Chao1 index as well as Observed species had a highly significant positive correlation (*p* < 0.01).

#### 3.3.2. Redundancy analysis of environmental factors and soil bacterial and fungal communities

**Figure 6:**
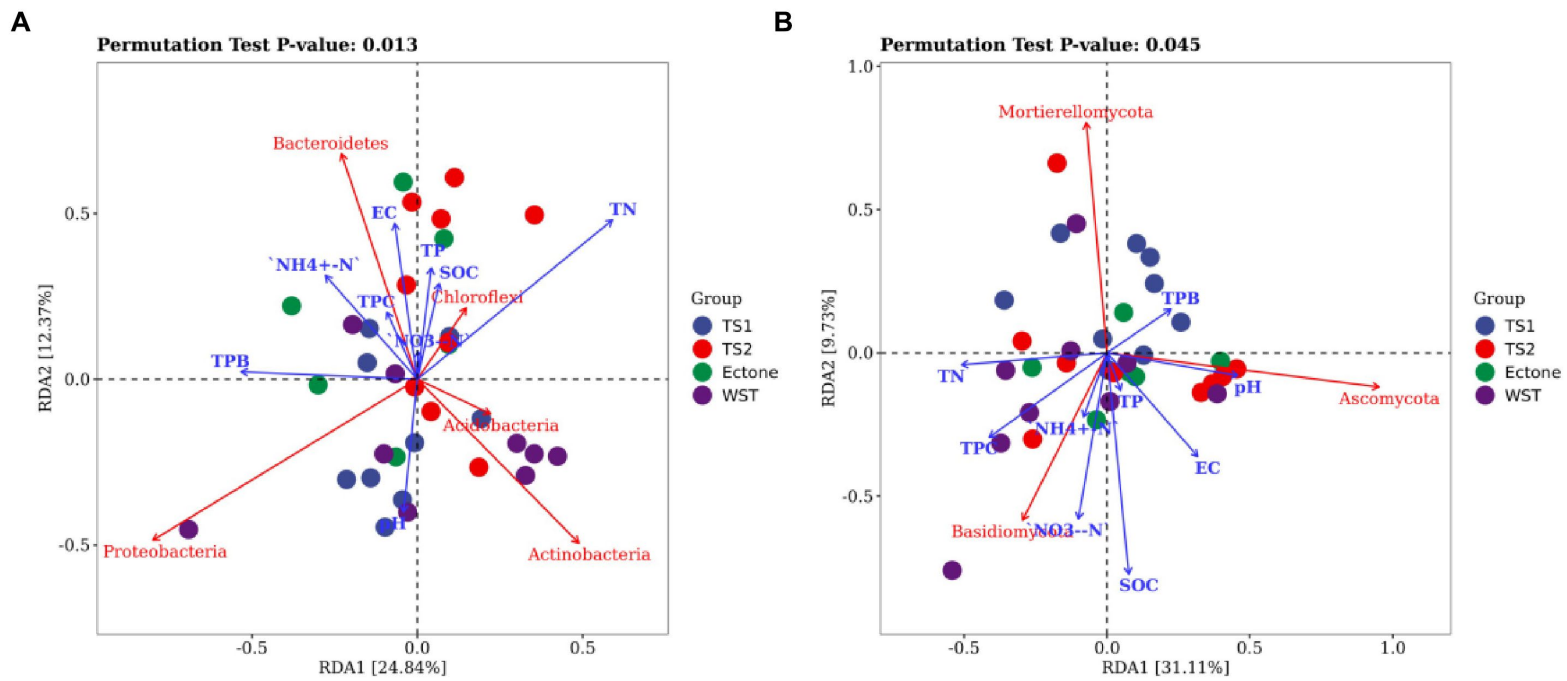
RDA analysis between **(A)** bacterial and **(B)** fungal communities and environmental factors. TN represents total nitrogen; TP represents total phosphorus; SOC represents soil organic carbon; EC represents electrical conductivity; NH4^+^-N represents ammonium nitrogen; NO3^−^-N represents nitrate nitrogen; TPC represents total plant cover; TPB represents total plant biomass; TS1 represents temperate steppe-sandy soil; TS2 represents temperate steppe-loamy soil; Ecotone represents warm and temperate ecotone; WST represents warm-temperate shrub. The sample size of TS1, TS2, and WST is 9, and the sample size of Ecotone is 6.

RDA mainly reflects the relationship between microbial communities, samples, and environmental factors using two-dimensional planes through the way of dimensionality reduction. As shown in Figure 6, the cumulative explained variance of the 9 environmental factors on the changes of bacterial and fungal communities structure reached 24.84, 12.37 and 31.11%, 9.73% for the 1st and 2nd ranking axes of RDA analysis, respectively, indicating that the first 2 ranking axes could better reflect the relationship between soil bacterial community structure and basic physicochemical properties of soil, and were mainly determined by the 1st axis.

The results showed that soil total nitrogen content, pH and conductivity were important factors influencing the changes of bacterial communities in different grassland soil types. And soil total nitrogen content was positively correlated with the relative abundance of *Chloroflexi* and negatively correlated with that of *Proteobacteria*. Soil pH was positively correlated with the relative abundance of *Proteobacteria* and *Acidobacteria*, and negatively correlated with the relative abundance of *Chloroflexi* and *Bacteroidetes*. Moreover, soil organic carbon, total nitrogen content and conductivity were important factors influencing the change of fungal communities in different grassland soil types. And soil organic carbon content and conductivity were positively correlated with the relative abundance of *Basidiomycota*, and negatively correlated with the relative abundance of *Mortierellomycota*.

## 4. Discussion

*Actinobacteria* and *Ascomycota* were the common dominant phylum of bacterial and fungal communities in different grassland soil types in northwest Liaoning (Figure 3). This is similar to the structural composition of fungal communities in Hulunbeier sand area, Loess Plateau grassland and Inner Mongolia desert grassland (Huang et al., 2021), but there are obvious differences with the structural composition of their bacterial communities, probably because the grassland in northwest Liaoning is in the process of recovery. *Actinomycetes* have a variety of antibacterial, antioxidant and enzyme inhibitor functions (Amrita et al., 2012; Arumugam et al., 2017), and they have positive effects on both soil and plants in grassland, which can change the dominant flora and eliminate harmful microorganisms, while protecting and promoting root growth. And *Ascomycota* have the function of degrading environmental pollutants and restoring soil (Germain et al., 2021), while most of the grasslands in northwest Liaoning are degraded grasslands, which can explain the dominant role of *Ascomycota* in this study. Moreover, there may be a close link between the relative abundance of soil bacterial community genera and their functions, soil physicochemical properties may be an important factor influencing the differences in bacterial community composition (Liang et al., 2021). *Subgroup 6* plays an important role in the global carbon cycle (Pan et al., 2019), and their relative abundance correlates with soil carbon content. In this study, the relative abundance of *Subgroup 6* of temperate steppe-sandy soil was significantly lower than that of other grassland soil types, while the soil organic carbon content was similarly significantly lower than that of other grassland soil types (Table 2). The relative abundance of *RB41* is somewhat representative of inter-root soil nutrient changes, and there is a significant positive correlation between them (Liu et al., 2022). The soil nutrient content of temperature step sandy-soil in this study is the lowest (Table 2), which explains why its relative abundance of *RB41* is significantly lower than that of other grassland soil types. *Rubrobacter* is highly resistant to radiation, salinity, heat and even thermophilicity, and soils with higher relative abundance of *Rubrobacter* are more resistant to stress (Kouřilová et al., 2021). In the study, the relative abundance of *Rubrobacter* in temperate steppe-sandy soil was significantly lower than in other grassland soil types, indicating that temperate steppe-sandy soil had the least resistant. In this study, most of the soil fungal community genera contained high virulence and pathogenicity, such as *Fusarium* and *Penicillium*. The reason may be the degradation of the sampled grassland soils. *Pseudogymnoascus* belongs to cold-tolerant fungi, which are more suitable for survival in alpine environments (Shi et al., 2021), which explains why he relative abundance of *Pseudogymnoascus* was the highest in temperate steppe-sandy soil.

Meanwhile, the more connections in the soil microbial community network, the higher its stability and the stronger the ability to inhibit the invasion of pathogens (Shi et al., 2016; Wei et al., 2018). In this study, the degree of connection in the soil bacterial network of grassland was much higher than that in the fungal network (Figure 4), indicating that the bacterial community had a strong resistance to invasion, which further proved that the bacterial community dominated the grassland in northwest Liaoning. In addition, the extent of grassland degradation or restoration can be predicted to some extent by soil microbial symbiotic networks. Studies have shown that enhanced microbial interactions and higher levels of recovery in grasslands lead to more complex interaction networks (Gao et al., 2021). The microbial symbiotic network in this study was relatively simple and belonged to a degraded grassland (Figure 4).

Microbial diversity has an important influence on the stability of grassland ecosystems, and the microbial diversity index can evaluate the abundance and evenness of microbial communities in soils, as well as reflect the different species composition and functions of microbial communities (Kim et al., 2017). Alpha diversity of bacterial and fungal communities in different grassland soil types showed significant differences in the study of Liu (2019), which is consistent with the findings of this study. The abundance, number of OTUs, number of species and diversity of both soil bacterial and fungal communities in the warm and temperate ecotone were all the highest (Figure 1). The reason may be that the warm and temperate ecotone is simultaneously influenced by both warm and temperate grassland types, with a large environmental gradient and high primary and secondary productivity resulting in a rich diversity of soil bacterial communities in grasslands. Which is reflected in Huang et al. (2016), where microbial diversity was enriched under the influence of dual environmental factors. However, Araujo et al. (2021) reported that the soil nutrient content and microbial diversity index in the interlacing zone showed intermediate values of adjacent communities. Ecotones are areas where two biomes meet due to sudden changes in soil properties caused by anthropogenic activities or changes in climatic conditions, and are areas of very high species abundance and diversity (Eddy, 1990; Bossuyt et al., 1999). Most studies on ecotones have focused on plants, animals and insects, and ecological ecotones are usually identified by obvious changes in above-ground vegetation (Pinheiro et al., 2015; Coelho et al., 2017). Nevertheless, there is no clearer understanding of soil microbial properties, so the study of microorganisms in ecotones needs to continue to be explored continuously. In addition, the bacterial diversity index of warm-temperate shrub was almost significantly lower than that of other grassland soil types (Figure 1). The reason may be that the presence of shrubs makes the grass covered by stunted growth and development, which in turn affects the diversity of bacteria. In Idbella’s study (Idbella et al., 2022), it was demonstrated that the abundance of soil oligotrophs under shrub cover decreased significantly, which in turn decreased the soil bacterial diversity.

At the same time, there were also obvious differences in microbial community structure among different grassland soil types (Figure 2). The structure of both bacterial and fungal communities in temperate steppe-sandy soil differed significantly from other grassland soil types (Supplementary Figure S2). In the soil environment of the whole Liaoning Province, the sand content is relatively high, which is due to the desertification caused by soil degradation. We divide the temperate steppe into two soil types (sandy soil and loamy soil) according to soil texture and properties, while the warm and temperate ecotone and the warm-temperate shrub are both loamy soil, which makes temperate steppe-sandy soil is absolutely unique. In the study of Sessitsch et al. (2001), changes in soil particle size lead to changes in soil microbial community structure, which could explain the significant differences of microbial community structure between temperate steppe-sandy soil and other grassland soil types. In addition, studies have shown that there were significant differences in bacterial and fungal community structure during the succession stages of grassland degradation and restoration (Guo et al., 2019), which means that the degree of degradation of grassland soil can be judged by observing the differences in microbial community structure of different grassland soil types. In this study, the microbial community structure was significantly different in the temperate steppe-sandy soil (Figure 2), which proved the highest degree of soil degradation.

Environmental factors were strongly associated with soil microbial communities (Franklin and Mills, 2009). The Pearson correlation analysis showed that the diversity of soil fungal communities was positively correlated with plant cover and negatively correlated with soil pH (Figure 4). As the most important characteristic of soil, pH could affect the diversity of soil microorganisms by altering their nutrient utilization efficiency and enzyme activity. In the study by Zhang et al. (2017), higher soil pH could reduce the nutrient use efficiency of fungi, which in turn reduced the diversity of the fungal community. In addition, there was a correlation between soil pH and soil salinity, and a joint increase in both also inhibited microbial activity (Yang et al., 2020). Besides, there was a positive correlation between plant cover and soil phosphorus content, and microbial assimilation and storage of P was fundamental for its availability for plant colonization (Canini et al., 2019). In the study by Dang et al. (2018), the increase of plant cover improved soil ecological functions and optimized the nutrient environment, which in turn increased fungal diversity. However, the Alpha diversity of soil bacterial communities was not restricted by the measured environmental factors in spite of the significant differences in bacterial diversity among different types. What’s more, it can be seen that the responses of soil bacterial and fungal communities to environmental changes were inconsistent, this is supported by the different responses of soil bacterial and fungal communities to different nitrogen application levels and pH intervals in the study by Li et al. (2019).

The RDA analysis showed that the environmental factors affected the soil bacterial and fungal communities, which was consistent with the finding of Furtak that microbial communities with higher relative abundance are significantly affected by environmental factors (Furtak et al., 2020), but the dominant influencing factors would be different. In the present study, soil total nitrogen content, organic carbon, pH and electrical conductivity were found to be main factors explaining the changes observed in the microbial community structure in grasslands of northwest Liaoning. Soil total nitrogen content has multiple effects on the growth, composition and function of microorganisms (Zhang et al., 2018). Soil pH could shape microbial community structure by modifying enzymes activity and controlling the accessibility of nutrient and moisture supplements (Cao et al., 2016; Ren et al., 2018). Soil electrical conductivity affects the conversion and cycling of phosphorus and carbon in the soil, which in turn influences the microbial communities (Nan and Petra, 2013). The key parameter of soil organic carbon cycling is microbial carbon use efficiency, and its content significantly affects the activity of soil microorganisms (Wu et al., 2022). What’s more, RDA ordination revealed distinct differences in microbial community composition between grassland soil types. Microbial communities of the temperature steppe-sandy soils tended to be distributed in environments with lower soil total nitrogen and organic carbon contents (Figure 6), which illustrated the lowest soil properties in the temperature steppe-sandy soils (Table 2). However, the environmental factors selected in our study were not enough to explain the structural changes in soil bacterial and fungal communities in northwest Liaoning grasslands. Soil microbial communities could also be influenced by soil salinity, climate, season, and geographic location factors (Liang et al., 2022). Furthermore, light may affect carbon cycling and thus influence microbial communities through plant photosynthesis, and microbial communities would be also strongly influenced by temperature (Ruiz and Azcon, 1996), and there may be interaction effects between the various factors (Yao et al., 2018). Therefore, a wider range of environmental factors should be further investigated in the future to obtain more comprehensive information. Soil total nitrogen and organic carbon content are considered important indicators of grassland quality (Wang et al., 2022), and studies have shown that soil quality in degraded grassland is severely deteriorated and soil total nitrogen and organic carbon content was significantly reduced (Byrnes et al., 2018). Yang (2022) also demonstrated that soil carbon and nitrogen levels gradually increased as grasslands were restored. In this study, the temperate grassland sandy soil had the lowest total nitrogen content and significantly lower organic carbon content than other grassland soil types, which also proved that it had the highest degree of grassland degradation.

## 5. Conclusion

In summary, our results implied that the structural composition and diversity of both bacterial and fungal communities in different grassland soil types in northwestern Liaoning Province were significantly different, but bacterial and fungal communities differed in their responses to the environment and bacterial community network was more stable than fungal community network. Temperate steppe-sandy soils were the most degraded grasslands. The diversity of bacterial and fungal communities was highest in the warm and temperate ecotone soils, while the warm-temperate shrub soils had the lowest bacterial diversity. The microbial community structure of temperate steppe-sandy soil differed significantly from the others. Our findings revealed the crucial influence of soil pH and plant cover on fungal community diversity and suggested that soil total nitrogen content, pH, electrical conductivity and soil organic carbon content predominantly explained the variability of soil microbial community structures. Our study demonstrated that changes in grassland ecosystem health can be predicted to some extent by microbial community structure and the corresponding observations of soil total nitrogen and organic carbon content.

## Data availability statement

The datasets presented in this study can be found in online repositories. The names of the repository/repositories and accession number(s) can be found in the article/Supplementary material.

## Author contributions

BR, LB, and JL designed this study. XM, JY, JW, DL, and MM performed the laboratory analysis. XM and BR wrote the paper. All authors contributed to the article and approved the submitted version.

## Funding

This study was supported by National Natural Science Foundation of China (32001127) and Science and Technology Plan of Liaoning Province (2020JH1/10300006).

## Conflict of interest

The authors declare that the research was conducted in the absence of any commercial or financial relationships that could be construed as a potential conflict of interest.

## Publisher’s note

All claims expressed in this article are solely those of the authors and do not necessarily represent those of their affiliated organizations, or those of the publisher, the editors and the reviewers. Any product that may be evaluated in this article, or claim that may be made by its manufacturer, is not guaranteed or endorsed by the publisher.

## Figures and Tables

**Table:** 

TS1	temperate steppe-sandy soil
TS2	temperate steppe-loamy soil
Ecotone	warm and temperate ecotone
WST	warm-temperate shrub
TPC	total plant cover
TPB	total plant biomass
QIIME	Quantitative Insights into Microbial Ecology
ANOVA	One-way analysis of variance
PCoA	principal coordinate analysis
Anosim	Analysis of similarities
RDA	Redundancy analysis
TN	soil total nitrogen
TP	soil total phosphorus
SOC	soil organic carbon
EC	soil electrical conductivity
NH_4_^+^-N	soil ammonium nitrogen
NO_3_^—^N	soil nitrate nitrogen
OTUs	operational taxonomic units

## Footnotes

^1^https://docs.qiime2.org/2019.4/tutorials/
