# Supplementary material for: Changes in grassland soil types lead to different characteristics of bacterial and fungal communities in Northwest Liaoning, China
Source: Front Microbiol. 2023 Jun 28;14:1205574. doi: 10.3389/fmicb.2023.1205574 (PMC10336218; doi:10.3389/fmicb.2023.1205574)
Supplement: Supplementary file 1 [file Image_1.pdf]

# Changes in grassland soil types lead to different characteristics of bacterial and fungal communities in northwest Liaoning, China

**Xinwei Ma[1], Baihui Ren[1*] Jianxin Yu[1], Jiayu Wang[1], Long Bai[1], Jiahuan Li[1], Daiyan Li[1], Meng Meng[1]**

[1]Shenyang Agricultural University, Shenyang Liaoning 110866, China

***Correspondence:** Baihui Ren: Email address: bhren@syau.edu.cn

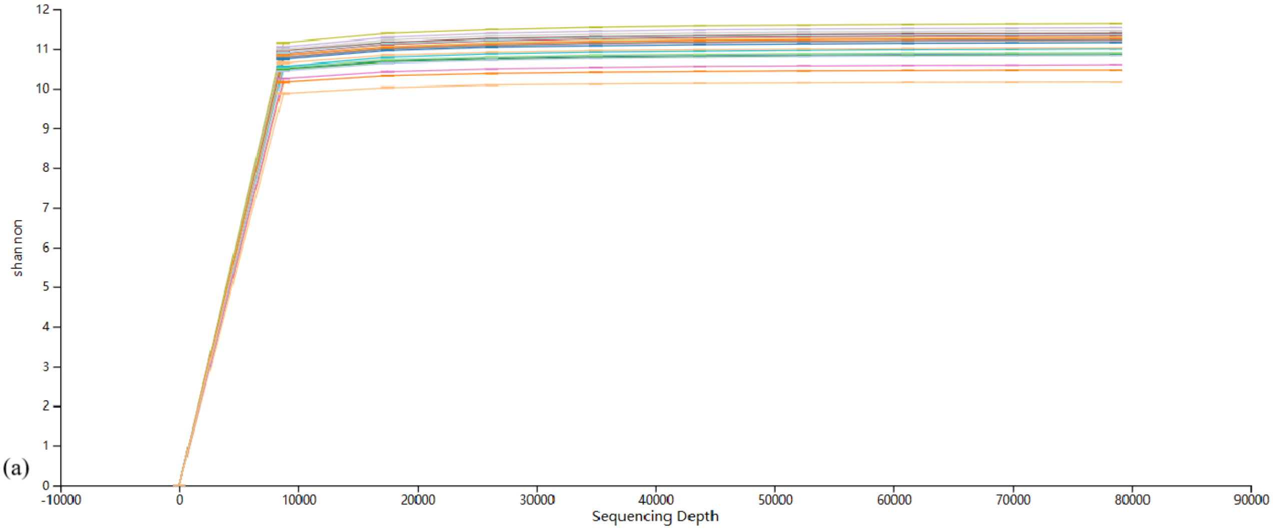


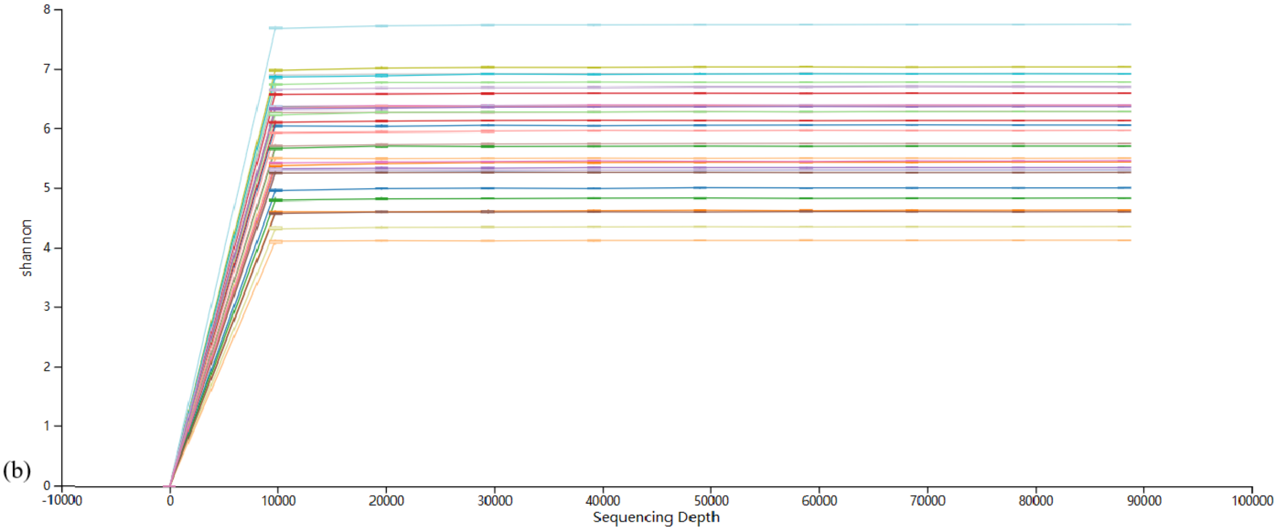


Figure S1 Rarefaction curves of (a) bacterial and (b) fungal communities

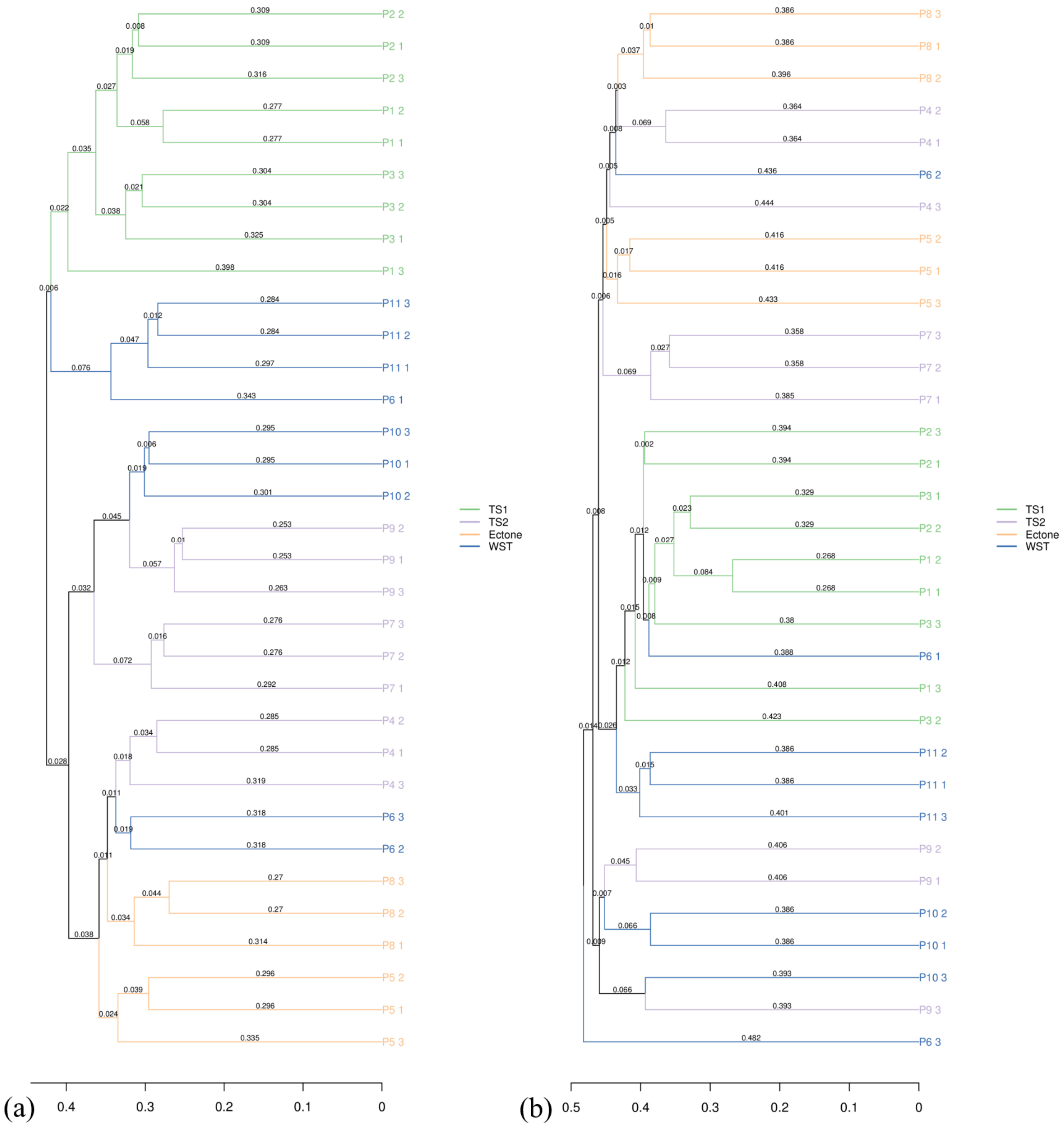


Figure S2 Cluster analysis of (a) bacterial and (b) fungal communities in different grassland soil types. TS1 represents temperate steppe-sandy soil; TS2 represents temperate steppe-loamy soil; Ecotone represents warm and temperate ecotone; WST represents warm-temperate shrub.
